# Feasibility of setting up a pre-operative optimisation ‘pre-hab’ service for lung cancer surgery in the UK

**DOI:** 10.1186/s13741-020-00145-5

**Published:** 2020-05-13

**Authors:** William M. Ricketts, Karen Bollard, Emma Streets, Kristi Hutton, Catherine Hornby, Kelvin Lau

**Affiliations:** 1 Barts Thorax Centre, London, UK; 2 Barts Cancer Centre, London, UK; 3 Barts Health NHS Trust, London, UK; 4 Charing Cross Hospital, London, UK

## Abstract

Pre-operative optimisation ‘pre-hab’ is a growing area in peri-operative medicine. This is usually undertaken with the aim of reducing post-operative complications. In the case of early-stage lung cancer, surgery is the treatment modality with the best-proven cure rates. With this in mind, we set up a pre-hab service, not merely to reduce the risk of post-operative complications, but to enable patients of borderline fitness for surgery to safely undergo this potentially lifesaving treatment. We believe this service to be one of the first of its kind in the UK, here we describe the challenges we faced in setting it up and the outcomes from our first 50 patients.

## Background

Lung cancer is the commonest cause of cancer death in the UK (Cancer Research UK 2018) with our region of North-East London historically having amongst the highest age-standardised incidence rates and lung cancer mortality in the country (Public Health England 2014). The treatment for lung cancer with the best-proven cure rates is surgery, with higher resection rates correlating with improved survival (Riaz et al. 2012). Accordingly increasing resection rates is advocated to improve lung cancer survival across the UK with guidelines suggesting that surgery should be offered wherever possible (Lim et al. 2010). However, the majority of patients with lung cancer present with advanced disease for which surgery is not an option (The Royal College of Physicians 2019).

The National Lung Cancer Audit (NLCA) has shown significant variation in surgical resection rates, with rates ranging from 10 to 37% in the most recent report, with similar variation in 1-year survival from 27 to 49% even when case-mix adjustment is applied (The Royal College of Physicians 2019). There is little data available on what drives this variation (Belot et al. 2019; Navani et al. 2018). The NLCA initially set a resection rate target of 16%, more recently this threshold was increased to 17%. In the most recent iterations of the audit, data was also published on rates of ‘treatment with curative intent’ for patients with early-stage disease and good physical fitness, highlighting that only 60.7% of patients with early-stage disease were being treated surgically with a further 20.1% receiving other forms of curative treatment such as radical radiotherapy, again with significant variation from 50 to 100% (The Royal College of Physicians 2019).

The NLCA audit data for 2014 showed that our hospital, despite being a thoracic surgical centre, had a resection rate of 12.1%, significantly lower than the national average even when allowing for case-mix adjustment (The Royal College of Physicians 2015). In response to this, the case notes of all early-stage patients in our Trust were reviewed for the years 2014 and 2015 with the single most commonly cited reason for not undergoing surgery being respiratory comorbidities (38.7%), with cardiac comorbidities (22.6%) also making up a significant proportion (Ogunsanya et al. 2017).

Based upon the hypothesis that respiratory function could be improved by optimising treatment for underlying comorbidities, most commonly chronic obstructive pulmonary disease (COPD), potentially tipping the balance between a patient being deemed not fit enough for surgery and being considered a surgical candidate, a pre-operative optimisation ‘pre-hab’ programme was initiated. We believe this to be one of the first such lung cancer programmes in the UK, here we describe the challenges we encountered and the outcomes of our first 50 patients, presented in the context of a feasibility study as described by Orsmond and Cohn (2015).

1. Evaluation of recruitment capability and sample characteristics
2. Evaluation and refinement of data collection procedures and outcome measures
3. Evaluation of acceptability and suitability of intervention and study procedures
4. Evaluation of resources and ability to manage and implement the study intervention
5. Preliminary evaluation of participant responses to intervention

## Case presentation

### Evaluation of recruitment capability and sample characteristics

The British Thoracic Society recommends the assessment of fitness for surgery based on cardiovascular, respiratory and overall mortality risks with baseline lung function testing used to guide the respiratory component (Lim et al. 2010). Our aim was to increase our resection rate by targeting those who were felt to be of borderline fitness and offering them pre-hab. Given that the evidence base for predicting post-operative breathlessness is limited and the correlation between self-reported exercise capacity and formally measured exercise capacity is moderate at best (National COPD Audit Programme: pulmonary rehabilitation workstream 2013–18 2018) deciding who to refer to this limited resource proved challenging. With no formal criteria devised, the decision to refer was based upon individual clinician clinical reasoning. In the early stages of the service, clinician buy-in was a barrier to referral, but following championing from a handful of clinicians and the benefits becoming more apparent; this has improved with a wider range of clinicians now referring.

In 2014, there were 338 patients treated for lung cancer across our Trust with 132 of those at our site (The Royal College of Physicians 2015) with our baseline audit suggesting that approximately 15 patients per year at our site and 40 per year across the Trust were potentially operable but not being treated surgically, in up to 40% of these cases this was due to respiratory comorbidities (Ogunsanya et al. 2017). Uptake has not been formally audited, but no patients that were referred declined and with 50 patients recruited over almost exactly 4 years, this would suggest that enrolment at our site has been roughly in line with anticipated demand, but there have been less referrals than might be expected from the other sites in the Trust, perhaps due to less buy-in and other barriers such as travel and perceived accessibility.

The baseline characteristics of our first 50 patients are described in Table 1, with 78% having at least moderate COPD and a mean FEV1 of 1385 ml (59.5% predicted) and diffusion coefficient for carbon dioxide (DLCO) of 54.6% predicted with a mean baseline Medical Research Council (MRC) dyspnoea score of 3.75 (range 2-5).

**Table 1:** Baseline characteristics of pre-hab cohort

Demographic	Cohort characteristics
Gender	Male 35 (70%)
Age (mean; range)	72.68 (55-88)
COPD status	
• None	2 (4%)
• Mild (FEV1 ≥ 80% predicted)	9 (18%)
• Moderate (FEV1 50-80% predicted)	17 (34%)
• Severe (FEV1 30-50% predicted)	19 (38%)
• Very severe (FEV1 < 30% predicted)	3 (6%)
On optimal inhalers at initial review (*n* = 46)	21 (45.7%)
Smoking status	
• Current	20 (40%)
• Ex	25 (50%)
• Never	3 (6%)
• Not recorded	2 (4%)
Baseline FEV1 (ml) (mean; IQR)	1,385 (880-1730)
Baseline FEV1 (% predicted) (mean; IQR)	59.5% (35.8-74.8%)
Baseline DLCO (% predicted) (mean; IQR) (*n* = 45)	54.6% (42-66%)
Self-reported baseline exercise tolerance (m) (mean; IQR) (*n* = 28)	254.6 (31.3-262.5)
Baseline MRC dyspnoea score (mean; IQR) (*n* = 43)	3.74 (3-5)

DLCO diffusion coefficient for carbon monoxide, FEV1 forced expiratory volume in 1 s, IQR interquartile range, MRC Medical Research Council

Although we were unable to undertake pre- and post-pre-hab cardiopulmonary exercise testing (CPET) when utilised, it still provided us with useful data, including highlighting the poor correlation between self-reported and objective exercise capacity. CPET testing also highlighted that despite their predominantly respiratory comorbidities, not all patients were respiratory limited when tested formally. Of the 19 patients to undergo CPET testing, six each (32%) showed predominantly respiratory limitation or no significant limitation, four (21%) showed deconditioning, with two (11%) showing a mixed picture and one (5%) being cardiac limited. With more data, we may be able to identify whether any of these sub-groups respond better to pre-hab.

### Evaluation and refinement of data collection procedures and outcome measures

Routinely recorded outcome measures such as hospital length of stay and post-operative complications were recorded. Where possible, lung function testing was repeated prior to surgery, but the opportunity to do this was often hampered by the short notice at which operation dates were confirmed. Ideally, cardiopulmonary exercise testing (CPET) would have been undertaken at initial assessment and repeated on completion of the pre-hab programme to assess baseline fitness, the impact of the programme and surgical risk. However, as CPET is a limited resource, control over the timing was not possible and often fell in the middle of the programme with insufficient capacity to allow repeat testing.

In addition to these routinely collected metrics, we felt it was important to record some specific outcome measures, both with a view to collecting evidence for the programme, but more importantly, to be able to assess individual patient’s progress. Evidence-based outcome measures were chosen to demonstrate physical, qualitative and cost-effective improvements as follows:

- The 6-min walk test (6MWT)—commonly used in this patient group to assess physical fitness. This was chosen due to the ease of administration and the ease for patients to complete (Temel et al. 2009).
- Five times sit-to-stand (FTSTS)—a quick and effective way of measuring a functional movement with the ability to demonstrate small improvements (Whitney et al. 2005).
- EuroQol five dimensions five-level (EQ-5D-5 L)—a measure of health-related status (5 dimensions: mobility, self-care, usual activities, pain/discomfort and anxiety/depression) developed by the EuroQol group providing a measure of health states that can be converted into a single index value facilitating a calculation of quality-adjusted life years (QALYs) (Herdman et al. 2011).

These outcome measures were completed on initial assessment and opportunistically at follow-up appointments. A final assessment before surgery was not always possible as operation dates were often confirmed at relatively short notice. Prior to settling on these outcome measures, we trialled alternatives including 10-metre walk speed and incremental shuttle walk tests, as functional measures, and the 12-item short-form health survey (SF-12) as a quality of life measure, but settled on those three due to simplicity of administration and analysis alongside the validated nature of the outcome measures.

### Evaluation of acceptability and suitability of the intervention

#### Nature of programme and safety

In the baseline audit, the majority of patients were deemed inoperable due to respiratory comorbidities, as such the constituents of the programme were based around the mainstays of COPD management:

- Optimising inhaled therapy
- Smoking cessation and
- Pulmonary rehabilitation

For those patients limited by pure cardiac comorbidities or combined respiratory and cardiac comorbidities, access to cardiac services was provided by our hospital’s cardio-oncology service.

COPD is common in patients with lung cancer due to the shared risk factor of cigarette smoking and patients being investigated for lung cancer should undergo spirometry, with those being considered for radical treatment also undergoing gas transfer assessment (Lim et al. 2010). Thus, by ensuring all patients referred undergo lung function testing as part of their initial assessment, previously undiagnosed or sub-optimally treated COPD is frequently uncovered, providing the opportunity to optimise COPD management early in the patient pathway. From our initial cohort of 50 patients, there was a scope to optimise inhalers in 25 of the 46 (54.4%) who met criteria for inhaled therapy.

Similarly, smoking status is also routinely assessed in the clinic, with 20 patients in the cohort (45%) being current smokers of whom eleven (55%) reported successfully quitting prior to their surgery. Both our unpublished work (presented at the Prehabilitation World Congress 2019) and that of others suggest that surgery is a teachable moment in smoking cessation enhancing quit rates (NIHR Cancer and Nutrition Collaboration et al. 2019; Shi and Warner 2010).

The British Thoracic Society (BTS) guidelines recommend referral for pulmonary rehabilitation (PR) for all patients with COPD and a MRC dyspnoea score of two or more, with grade A evidence for those with a score of three or more (Bolton et al. 2013). All but two patients in our cohort met these criteria, both due to the absence of COPD on spirometry, both confirming that basing our programme around the mainstays of COPD management appeared a sensible approach and also that the principles of pulmonary rehabilitation should underpin the exercise component of the programme.

In keeping with the BTS PR guidelines (Bolton et al. 2013), exercise programmes were based around progressive muscle resistance and aerobic training. Dependent on patient choice, patient need and availability these programmes were either one-to-one or group sessions led by our oncology outpatient physiotherapy team, standard PR led by our local community respiratory team or intensive inpatient prehabilitation led by our cardio-respiratory physiotherapy service. Some patients underwent a hybrid approach with initial review by an Oncology Outpatient Physiotherapist, with on-going review either by telephone follow-up or as part of local PR. All patients were seen post-operatively, as per the standard protocol, and discharged from the inpatient physiotherapy service once all inpatient goals were achieved. All patients were offered onward referral to local PR classes on discharge.

There were no safety issues reported in any cohort of patients with the median length of stay for all patients undergoing lung cancer surgery in our institution of 6 days (IQR 4-9), which is identical to the national average, with those in the outpatient pre-hab cohort having a median length of stay of 8 days (IQR 6-16). There was only one death in our surgical cohort, giving both a 30 and 90 day mortality of 2.9%, which is similar to the national averages of 1.8% and 3.5% mortality, respectively (The Royal College of Physicians 2018). None of these differences are statistically significant, but even had they been it would not be surprising given that these patients are chosen for pre-habilitation based upon their high peri-operative risk. This suggests that patient selection is broadly appropriate and that pre-hab is safe in this context, possibly even successfully attenuating that risk, although larger numbers are required.

#### Duration of programme

Where our programme had to deviate from standard PR was in the duration of the programme, with the BTS guideline recommending at least 12 sessions for 6-12 weeks, this was not feasible in the context of a 62-day lung cancer pathway. Reviewing the pre-hab specific literature gave highly variable programme durations ranging from as little as three days (Gao et al. 2015) to as long as a median of 8.6 weeks (Peddle et al.). However, since we initiated our programme both a systemic review and Cochrane review have been published (Cavalheri and Granger 2017; Steffens et al. 2018). The studies included in the systematic review describe one to two week programmes in lung cancer, but describe longer programmes in other tumour groups, with no specific comment on optimum duration. Indeed none of the studies included were designed to compare different durations of programme (Steffens et al. 2018). To achieve entry into the Cochrane review, the programme had to include at least seven sessions over at least one week, with the programmes included ranging from three sessions/day for one week to five sessions per week for four weeks, but again none of the studies directly compared one programme duration with another (Cavalheri and Granger 2017).

For the majority of the patients enrolled in our pre-hab programme, the duration was driven by the pragmatic constraints of the 62-day lung cancer pathway. For the 35 seen purely by the Outpatient Oncology Physiotherapy team the median number of sessions was three (range 1-7 sessions) over a median of 22 days (IQR 8-43 days). Eight patients (16%) underwent inpatient pre-hab, with a median duration of eight days (range 2-13 days) with this variance predominantly driven by scheduled operation date and agreement by individual surgeons as to whether this could be delayed.

For a small number of patients who started with a very low level of cardio-respiratory fitness or who required more extensive surgery and were making good progress with pre-hab a 62-day breach was accepted in order to further optimise them with the aim of reducing their peri-operative risk. Repeating the physiological outcome measures of 6MWT and FTSTS at each visit allowed us to assess which patients were still improving and which may have plateaued be that for physiological or motivational reasons.

To ensure that patients received the maximum duration of pre-hab, they were all referred as early as possible in the diagnostic pathway. However, with the benefit of hindsight, this led to some unnecessary or inappropriate referrals for 13 patients (26%) who later turned out to have either benign (3) or advanced-stage disease (5) with five further patients ultimately undergoing non-surgical treatment.

**Fig. 1:**
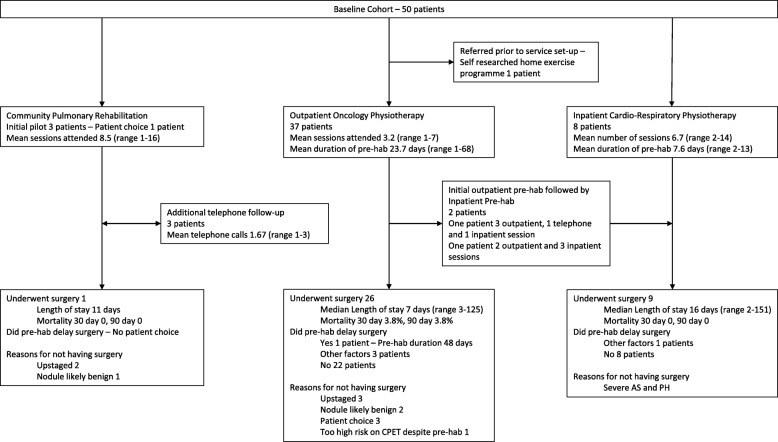
Consort diagram describing the nature of the pre-hab programme undertaken by each of the initial 50 patient cohort and their outcomes. AS, aortic stenosis; CPET, cardiopulmonary exercise test; PH, pulmonary hypertension

This pragmatic approach around location, nature and duration of programme as well as desire to refer early has led to significant variation in the nature of individual patient’s programmes driven predominantly by their own individual needs, this variation is summarised in Fig. 1.

#### Barriers to uptake

Despite the information given to patients and justification for the pre-hab programme given by their consultants, we experienced some difficulties with initial patient engagement. Some patients were reluctant to attend their initial assessment, feeling that this was unnecessary and not beneficial, making it difficult to book their appointments. Patients were often exhausted and frustrated by the number of appointments required for investigations combined with the shock of a lung cancer diagnosis. However, once they attended the first appointment, they usually understood the benefits and were happy to continue with the programme. To overcome this, wherever possible, physiotherapy appointments were booked on the same day as other appointments or investigations. Explanations and justifications were given to patients which also helped with their adherence and motivation.

Community pulmonary rehabilitation reduced travel for patients as they were able to undertake their pre-hab closer to home, but at the trade-off of less flexibility around timings of sessions. As we did not set a target number of sessions for patients to attend at the outset it is not possible to assess whether one format or another allowed improved adherence and this is something that should be looked at in the future.

No formal patient feedback was obtained, but informal feedback and high completion rates would suggest that the pre-hab programme was acceptable and appealing to patients. Furthermore, in a small sub-set of our cohort, we assessed quality of life using the EQ-5D-5 L which showed statistically significant improvements in the index values from 0.66343 to 0.80100 (*p* = 0.0213; 95% CI 0.02877-0.24637), although as data was only collected for the seven most recent participants it should be interpreted with caution.

Moving forward, we intend to collect formal patient feedback as well as produce a smartphone-based app to support the programme. This will facilitate the collection of patient-reported outcome measures (PROMs) to better evaluate the programme.

### Evaluation of resources and ability to manage and implement the intervention

The aim of our pre-hab programme was to increase our resection rate and, therefore, overall survival; however, despite the National Lung Cancer Audit setting a resection rate target, the target in itself attracts no financial incentive and, unlike NHS England’s 2-week-wait and 62-day treatment targets, neither does failing to meet it incur a fine, whereas all elements of the programme incurred some kind of cost. Furthermore, as pre-hab does not count as a definitive treatment, any additional time taken optimising patients for surgery takes time out of the 62-day pathway potentially risking a target breach fine.

Given that inhalers are a routinely funded component of COPD care for the vast majority of this cohort, and our smoking cessation clinic is funded by a local charity this left the exercise component of the programme as the only area requiring direct funding. As a tertiary oncology centre, an oncology outpatient physiotherapy service was already in place, but concerns were raised as to the unclear demand, finite capacity and short timescales involved. As almost all the patients meet standard criteria for pulmonary rehabilitation (PR), as a pilot and to help assess demand, we referred our early patients to our local PR service, negotiating for their referrals to be prioritised, such that they could attend the maximum number of sessions prior to their surgery (Bollard et al. 2017). Following the success of the initial PR based pilot, we were able to agree a further pilot with our outpatient oncology physiotherapy team with appointments funded as per standard outpatient physiotherapy tariffs.

Given that the aim of this project was to enable patients of borderline surgical fitness to undergo surgery, even in a large centre, this involves relatively few patients, with little outpatient physiotherapy capacity required, with a median of 13 patients per year requiring a mean of 3.2 sessions. We have discussed extending this pilot to fitter patients and/or those with advanced-stage disease at presentation and, therefore, not a candidate for radical treatment and are considering the role of a smartphone-based app in achieving this widening participation.

Although 26% of our cohort did not proceed with surgical treatment all of those with a proven cancer diagnosis did go on to receive some form of lung cancer treatment. It may be that pre-hab is also beneficial in the contexts of systemic therapy or radiotherapy, although the evidence is currently lacking, so these should not be viewed as superfluous referrals and this represents an area for future research.

With limited capacity, setting up a pre-habilitation service raises the issue of only being able to offer the programme to a sub-set of patients predicted as being most likely to benefit. In an ideal world, we would be able to assess all patients’ cardio-respiratory fitness with a field test such as a 6-min walk or incremental shuttle walk test at their first respiratory clinic appointment. This could be achieved relatively efficiently with the use of an appropriately trained technician. However, with only limited evidence on how to interpret these results in this context, this would only provide approximate guidance of who to refer on for full pre-hab via the specialist physiotherapy teams.

As a tertiary referral centre covering a large catchment area, some patients are unable to attend our Physiotherapy Outpatient Department regularly, if at all. Telephone follow-up has been shown to be feasible in this context (Granger et al. 2018), and we have piloted this successfully with a proportion of patients and are considering the role of digital solutions in further improving access.

### Preliminary evaluation of participant responses to intervention

Fifty patients undertook the pre-habilitation programme between 2015 and 2019. During this period the resection rate of the unit reported has improved from being a negative outlier at 12.8% to being a positive outlier at 29.8% (The Royal College of Physicians 2015, 2019). Whilst the small numbers that have been through the programme would not in themselves account for this shift, the pre-hab programme is an integral part of a wider culture change to improve radical treatment rates.

Data completeness has proved challenging in this feasibility study, predominantly due to difficulties in timing end of pre-hab assessments, but also evolving opinion on optimal outcome measures. However, significant improvements in physiological and quality of life measures were noted between the baseline and last available tests, with outcomes summarised in Table 2. Mean forced expiratory volume in 1 s (FEV1) (*n* = 24) improved by 14% (174 ml) from 1235 ml (50% predicted) to 1409 ml (57% predicted) (*p* = 0.0045, 95% CI 59.5-288.4 ml). Only nine patients completed a pre- and post post-pre-hab DLCO with a trend towards improvement (39.3% pre, 45% post, *p* = 0.0866, 95% CI − 1.02-12.36%).

**Table 2:** Outcome measures of prehab cohort

Outcome measure	Baseline mean (SD)	Change	*p* value
FEV1 (ml) (*n* = 24)	1235	+ 174	0.0045
DLCO (% predicted) (*n* = 9)	39.3	+ 5.7	0.0866
6MWT distance (m) (*n* = 25)	224.2	+ 81.5	< 0.0001
FTSTS (s) (*n* = 23)	27.5	− 9.6	0.0011
EQ-5D-5 L (*n* = 7)	0.66343	+ 0.13757	0.0213

*DLCO* diffusion capacity for carbon monoxide, *EQ-5D-5 L* EuroQol 5 dimension 5 level, *FEV1* forced expiratory volume in 1 s, *FTSTS* five times sit to stand, *6MWT* 6-min walk test

Mean five times sit to stand time (*n* = 23) improved by 36% (9.6 s) from 27.5 s to 17.4 s (*p* = 0.0011, 95% CI 4.3-14.9 s). Mean 6-min walk test (*n* = 25) distance improved by 36% (81.5 m) from 224.2 m to 305.7 m (*p* < 0.0001, 95% CI 46.5-116.5 m), significantly further than the minimally important difference in lung cancer of 22-42 m (Granger et al. 2015). Furthermore, although the numbers were small, the significant improvement in EQ-5D-5 L described above, would suggest an improvement in quality-adjusted life years. We were unable to complete both pre- and post-pre-hab CPET testing, giving no opportunity to assess for improvements in the more objective physiological parameters that this test provides.

Other pre-hab programmes describe the role of a dietician and possibly even a psychologist (Moorthy and Wynter-Blyth 2017). Anecdotally, it does not appear that there would be significant demand for this in our cohort, but formally assessing this need would be worthwhile.

## Discussion

Here we describe setting up one of the UK’s first lung cancer prehabilitation services with the hope that this can be replicated to improve resection rates and ultimately improve patient outcomes. This was an iterative process, but we believe it is feasible and that others can learn from our experience, with barriers and facilitators to this process summarised in Table 3.

**Table 3:** Barriers and facilitators to our prehabilitation service

Barriers	Facilitators
Patient selection	Lung function testing at initial outpatient appointment for all patients
Assessing demand	Piloted via standard PR before setting up bespoke oncology physiotherapy led service
Evidence base for elements of the programme	Based upon standard COPD optimisation (PR, smoking cessation and optimised inhaled therapy)
Funding	Utilised pre-existing resources (PR, oncology outpatient physiotherapy and smoking cessation clinic)
Duration of programme	Referrals made as early in the patient pathway as possible, with a target duration of at least 2 weeks
Choice of exercise programme	Mixture of aerobic and resistance exercises to moderate intensity, based upon standard PR
Accessibility	Telephone follow-up and/or local PR referral offered for those with difficulty attending. Use of a smart phone-based app proposed for future expansion.
Choice of outcome measures	Validated functional measures, 6-min walk test and 5 times sit to stand. Physiological tests FEV1 and DLCO; insufficient capacity to undertake pre- and post-pre-hab CPET testing. Quality of life measure with validated cost-effectiveness component EQ-5D-5 L.
Patient engagement	Service promoted by both chest physicians and thoracic surgeons with physiotherapist led telephone follow-up for non-attenders. Physiotherapy appointments scheduled to coincide with other appointments such as scans Telephone follow-up offered to enhance engagement.

*CPET* cardiopulmonary exercise test, *DLCO* transfer coefficient for carbon monoxide, *EQ-5D-5 L* EuroQol five dimension five level, *FEV1* forced expiratory volume in 1 s, *PR* pulmonary rehabilitation

In our service, we have pragmatically utilised a combination of one-to-one or group sessions led by specialist oncology outpatient physiotherapists, pulmonary rehabilitation and inpatient prehabilitation and hybrid combinations with or without telephone follow-up. We believe that this tailored approach has improved patient uptake, but at the expense of our ability to standardise either the exercise programme or the outcome measures. Further research is required on optimal nature and duration of exercise programmes as well as factors affecting patient adherence with their exercise programmes.

We have been fortunate that funding has been covered by existing services, but for other centres planning to set up a similar service negotiating rapid access to standard pulmonary rehabilitation may be easier to achieve than accessing specialist oncology physiotherapy services. The role of smoking cessation and optimising inhaled therapy should not be underestimated and, as standard care, should be cost-neutral.

Patient selection proved challenging, our capacity and the aim of improving resection rates meant that we targeted those felt most likely to benefit, but even identifying this cohort was not easy and it is likely that pre-hab would benefit a wider cohort of patients than we had a capacity for, with digital solutions being one option, whilst we are aware that others are utilising group sessions and community facilities.

Outcome measures have not been standardised in previously reported cohorts and we trialled various measures before settling on 6MWT, FTSTS and EQ-5D-5 L due to their simplicity of administration and analysis. These measures appear sensitive to change, demonstrated by both statistically and clinically significant improvements even in our relatively small cohort. We are aware of work elsewhere looking at standardising outcome measures across all pre-hab cohorts and we await the outcomes of this with interest.

Pre-hab in lung cancer patients based around the principles of COPD optimisation including pulmonary rehabilitation prior to surgery seems acceptable to patients and most importantly, given that this cohort of high-risk patients had outcomes similar to their lower risk peers, we think that this highlights that, with appropriate patient selection; pre-hab is safe and can help achieve improvements in resection rates.

The improvement in resection rates at our centre cannot be put down to pre-hab alone as the increase is greater than the number of patients that have been through our pre-hab programme. However, pre-hab has been part of a wider culture change including centralising our multidisciplinary team meeting (MDT), increasing our video-assisted thoracoscopic surgery (VATS) rate, undertaking more combined lung cancer resection and lung volume reduction surgery (LVRS) and investing in new technology such as a surgical robot and electromagnetic navigational bronchoscopy, as well as a wider enhanced recovery programme. It may be that pre-hab has also had a more tacit effect on physician and surgeon awareness around which patients previously deemed borderline are fit enough for resection without formally undertaking pre-hab themselves.

## Conclusions

The studies included in both the recent systematic review and the Cochrane review all utilise outcome measures of complication rate and length of stay (Cavalheri and Granger 2017; Steffens et al. 2018). Whilst these are clearly relevant, in setting up our programme, we argued that the priority was not reducing the already low risk of complications or shortening the already relatively short length of stay, but in enabling lifesaving surgery for those patients that would otherwise have been denied this treatment. However, despite the BTS guidelines describing assessment for fitness for surgery, the process remains relatively subjective and, with surgical techniques improving all the time, the true impact on resection rates of our pre-hab programme remains difficult to measure. However, we have shown the implementation of a lung cancer pre-hab programme to be feasible and safe and as part of a wider culture change, we have seen a significant improvement in our resection rates from being a negative outlier at 12.8% at the inception of this programme to being a positive outlier at 29.8% in the most recently available validated data (The Royal College of Physicians 2019).

Whilst there have been multiple hurdles to be overcome in setting up our pre-habilitation programme, given the significant improvement in our resection rate, we would recommend that this should be available to all lung cancer patients in the UK. This should be facilitated by an appropriate funding stream, as well as physiotherapy being recognised as ‘active treatment’ thus stopping or perhaps pausing the 62-day clock, at least in appropriately selected cases.

## Data Availability

The datasets during and/or analysed during the current study available from the corresponding author on reasonable request.

## Abbreviations

COPD: Chronic obstructive pulmonary disease
CPET: Cardiopulmonary exercise test
EQ-5D-5 L: EuroQol 5 Dimension 5 Level
FTSTS: Five times sit to stand
LVRS: Lung volume reduction surgery
MDT: Multidisciplinary team meeting
MRC: Medical Research Council
NLCA: National lung cancer audit
PR: Pulmonary rehabilitation
PROMs: Patient-reported outcome measures
QALY: Quality-adjusted life year
VATS: Video-assisted thoracoscopic surgery
